# Combined comparative genomic hybridization and transcriptomic analyses of ovarian granulosa cell tumors point to novel candidate driver genes

**DOI:** 10.1186/s12885-015-1283-0

**Published:** 2015-04-10

**Authors:** Sandrine Caburet, Mikko Anttonen, Anne-Laure Todeschini, Leila Unkila-Kallio, Denis Mestivier, Ralf Butzow, Reiner A Veitia

**Affiliations:** 1 Institut Jacques Monod, Paris, France; 2 Université Paris Diderot/Paris, Paris, France; 3 Department of Obstetrics and Gynecology, University of Helsinki and Helsinki University Central Hospital, Helsinki, Finland; 4 Children’s Hospital, University of Helsinki and Helsinki University Central Hospital, Helsinki, Finland; 5 Department of pathology, University of Helsinki, and HUSLAB, Helsinki University Central Hospital, Helsinki, Finland; 6 Université Paris-Diderot & Institut Jacques Monod, CNRS-UMR 7592, Bâtiment Buffon, 15 Rue Hélène Brion, Paris, Cedex 13 France

**Keywords:** Ovarian granulosa cell tumor, Driver genes, CGH, Transcriptomics

## Abstract

**Background:**

Ovarian granulosa cell tumors (GCTs) are the most frequent sex cord-stromal tumors. Several studies have shown that a somatic mutation leading to a C134W substitution in the transcription factor FOXL2 appears in more than 95% of adult-type GCTs. Its pervasive presence suggests that FOXL2 is the main cancer driver gene. However, other mutations and genomic changes might also contribute to tumor formation and/or progression.

**Methods:**

We have performed a combined comparative genomic hybridization and transcriptomic analyses of 10 adult-type GCTs to obtain a picture of the genomic landscape of this cancer type and to identify new candidate co-driver genes.

**Results:**

Our results, along with a review of previous molecular studies, show the existence of highly recurrent chromosomal imbalances (especially, trisomy 14 and monosomy 22) and preferential co-occurrences (i.e. trisomy 14/monosomy 22 and trisomy 7/monosomy 16q). In-depth analyses showed the presence of recurrently broken, amplified/duplicated or deleted genes. Many of these genes, such as *AKT1, RUNX1* and *LIMA1,* are known to be involved in cancer and related processes. Further genomic explorations suggest that they are functionally related.

**Conclusions:**

Our combined analysis identifies potential candidate genes, whose alterations might contribute to adult-type GCT formation/progression together with the recurrent FOXL2 somatic mutation.

**Electronic supplementary material:**

The online version of this article (doi:10.1186/s12885-015-1283-0) contains supplementary material, which is available to authorized users.

## Background

Ovarian granulosa cell tumors (GCTs) are the most frequent sex cord-stromal tumors, and account for more than 5% of ovarian cancers [1]. Two different forms, juvenile and adult, have been described based on the age of onset and histopathological features [2]. GCTs tend to be low-grade malignancies, but can recur up to 40 years after primary tumor resection [3]. Various studies have revealed that a somatic mutation leading to the p.C134W substitution in the transcription factor FOXL2 appears in > 95% of adult-type GCTs [4].

Transactivation studies have suggested that the p.C134W mutation could perturb the functional interaction between FOXL2 with SMAD3 [5] and FOXL2 activity in other systems [6]. This variant is also deficient in its ability to promote apoptosis [7] and displays a mild loss-of-function on targets involved in cell cycle and DNA-damage repair [8].

We have recently performed a transcriptomic profiling of 10 human adult-GCTs and ethnically-matched GC controls. This study showed that GCTs display several typical hallmarks of cancer. For instance, among FOXL2 direct targets, we detected an up-regulation of genes associated with cell cycle control and a down-regulation of genes related with apoptosis [9]. The pervasive somatic FOXL2 mutation is expected to be the main driver of GCTs. However, we hypothesize that it might engender or be accompanied by other mutations and genomic changes that might facilitate tumor formation and/or progression. Here, we have explored this possibility by performing a comparative genomic hybridization (CGH) analysis of the aforementioned tumor samples in correlation with their transcriptomes. This combined analysis is the first attempt to obtain a “bird’s eye” view of the genomic landscape of this cancer type and to identify new candidate (co-)driver genes (termed henceforth driver genes for simplicity).

## Methods

### Ethics statement

This research involves human samples and has been performed with the approval of the Ethics Committee of the Helsinki University Central Hospital. Research was carried out in compliance with the Helsinki Declaration.

### Comparative Genomic Hybridization (CGH)

The CGH was performed using genomic DNA from the tumor samples co-hybridized with an equimolar mix of 10 ethnically-matched (finnish) DNA samples on NimbleGen 12x135K CGH arrays, which 60-mer probes spaced every 13 kb on average. Sample processing, hybridization and data acquisition were performed at Nimblegen according to an in-house standard protocol. CGH microarray data are available in the ArrayExpress database (www.ebi.ac.uk/arrayexpress) under accession number E-MTAB-2873. CGH data were analyzed as log2 values of the ratio between the fluorescences of tumor and reference genomic DNA samples, using MeV software (TM4 suite, http://www.tm4.org).

### CGH and transcriptome correlations

For large-scale alterations, the CGH data were averaged for sliding windows of 130 kb over the relevant chromosomes. For the transcriptomic data, we used our previously published data from the 10 tumors, as described in [9] (NimbleGen Human Expression 12 × 135 K array set, accession E-MTAB-483 in the ArrayExpress database). The two independent transcriptomic hybridizations were averaged for each transcript, and then we computed the average expression levels for each gene.

To better measure the impact of large-scale genomic alterations on gene expression we divided the expression values for genes located within aneuploid regions by their mean expression in the tumors without the analyzed alteration. Expression ratios above/below 1 in the natural scale (or above/below 0 in log2 scale) in aneuploid regions are suggestive of a “correlation” between genomic duplications/deletions and gene expression. Finally, these ratios were averaged for 30 windows (of equal size) per chromosome. The CGH and transcriptomic profiles were displayed using MeV software.

To identify candidate drivers, we used the combination of several criteria. First, we aimed at identify genes with an expression correlated to small-scale imbalances. For this, the CGH probes, amplified/duplicated or deleted in at least 50% of the cells (which corresponds to log ratio of 0,322 or −0,415, respectively) and in at least two tumors, were identified using MeV software. Next, genes were retained for further analysis if one or several amplified/deleted CGH probe(s) mapped within 25 kb of the gene coordinates. Given the 13 kb-resolution of the CGH chip, a 25-kb maximum spacing enabled us to detect all relevant genes. Furthermore, transcriptomic values had to be significantly correlated with the CGH data over all the tumors (Pearson correlation coefficient, R). The threshold of statistical significance used for R was determined considering that, for a sample size **N**, with observed values of **R**, there is a statistic t such that:

$$ t=R\sqrt{\frac{n-2}{1-{R}^2}} $$

which follows approximately a Student-t distribution with **N**-2 degrees of freedom. Application of this formula to any particular observed value of **R** will test the null hypothesis that the observed value comes from a population in which the correlation between the two variables is 0. For a sample size of N = 10 (all tumors in our cohort), the R that can be considered as statistically significant according to this test is 0.63. In order to exclude any effect of large-scale imbalances (such as trisomies or monosomies), the gene-centered CGH/expression correlations were computed in the relevant genomic regions only for tumors without the large-scale imbalances. Therefore, we adjusted the threshold for R significance accordingly, to R > = 0.67 for 9 samples, R > = 0.71 for 8 samples and R > = 0.76 for 7 samples. Due to the small sample size of our cohort, we could not apply a correction for multiple testing, such as Bonferroni’s or Benjamini-Hochberg’s. Candidate driver genes were further selected when i) being completely included in the genomic alteration (i.e. fully amplified or deleted) and ii) not being included in frequent CNVs in healthy individuals, as defined by the Database of Genomic Variants (DGV, http://dgv.tcag.ca/dgv/app/home, as of 31/05/2013).

Recurrently broken genes were identified by the existence, in at least 2 tumors, of one or several closely mapping breakpoints defined by amplifications/deletions upstream *and* downstream, within the relevant gene. We excluded genes for which the breakpoints mapped near or within frequent CNVs according to DGV. This step was necessary because the control DNA used for CGH was a pool of ethnically-matching DNA samples, and not the somatic DNA from the respective patients.

### Expressional correlation, protein interactor sharing and transcriptomic neighbors sharing between candidate drivers

Hierarchical clustering of the expression levels of broken, amplified or deleted candidate drivers and *FOXL2* was performed with the MeV software, using complete linkage and the Pearson correlation coefficient as a measure of similarity.

For each candidate driver and *FOXL2*, two sets of transcriptomic neighbors were defined by a statistically significant correlation of expression in all tumors (R > = 0.63 or R < = −0.63). These gene sets were analyzed using the Enrichr tool (http://amp.pharm.mssm.edu/Enrichr [10]). The extensive sharing of transcriptomic neighbors between the candidate drivers or *FOXL2* was displayed using Cytoscape 3.0.1 software, keeping only the strongly correlated transcriptional neighbors (R > = 0,90) for clarity. The network was built using the “prefuse force directed” algorithm with EdgeBetweenness criteria, then manually edited for clarity.

## Results and discussion

### CGH of ovarian GCTs shows recurrent chromosomal imbalances

To identify DNA copy number changes in GCTs, we performed a CGH analysis of 10 tumor genomic DNA samples, using microarrays. All the tumors bear the FOXL2 somatic mutation C134W. Four tumors (H1, H8, H28 and H30) did not display any large-scale genome alterations. However, there was no obvious correlation between the absence of imbalances and tumor stage, size or age of occurrence. On the other extreme, the most altered tumor was H4, which is not surprising, owing to the fact that it is a recurrence (Additional file 1: Table S1a and S1b).

**Figure 1:**
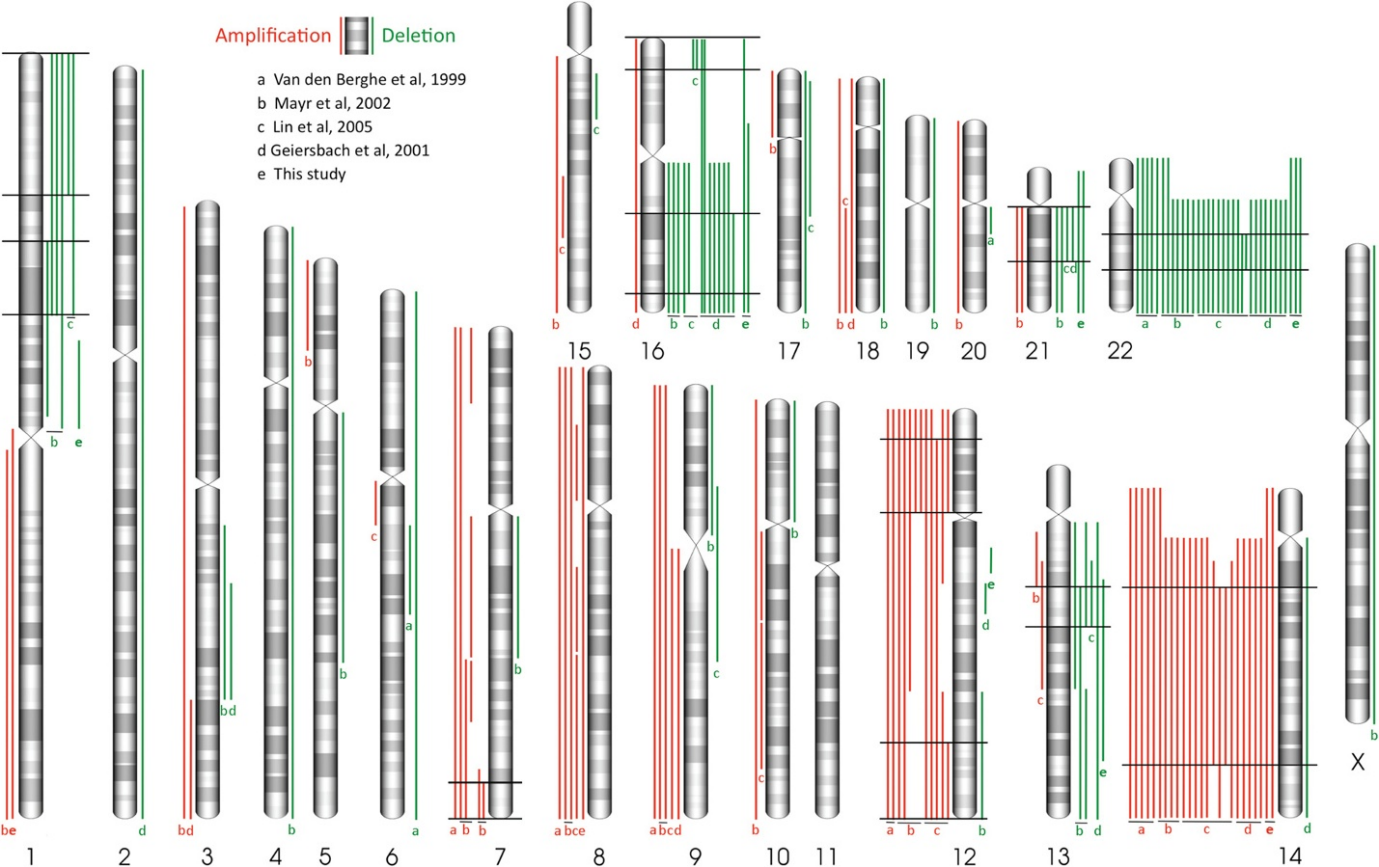
**Recurrent chromosomal imbalances in adult-type ovarian GCTs.** The CGH was performed using genomic DNA from the tumor samples co-hybridized with an equimolar mix of 10 ethnically-matched (finnish) DNA samples. Each chromosomal ideogram is depicted with amplifications in red (on the left) and deletions in green (on the right). This compilation includes data from 94 adult-type GCTs from 5 studies, among which 64 contain large-scale alterations. Smallest Regions of Overlaps (SROs), defined when several independent rearrangements point to a common altered genomic region, are likely to contain driver genes involved in tumor progression. Here, SROs are indicated (black horizontal lines) when they involve at least 5 imbalances of the same type (either amplifications or deletions). Details of chromosomal imbalances and co-occurrences identified by the five studies are provided in Additional file 1: Table S1c.

The detected large-scale imbalances were either recurrent or appeared only once in our samples. Whole-chromosome alterations involved trisomies 8 (1/10) and 14 (2/10), and monosomies 16 (1/10), 21 (2/10) and 22 (3/10). Other long-range changes included duplication of 1p11.1-qter (H4), and deletions of 1p11.1-p22.1 (H33), 12q13.11-q13.13 (H4), 13q13.3-q32.1 (H4), 16p11.2-qter (H4). Our analysis combined with a review of the literature ([11-14]) compiles the data of 94 adult-type GCTs (Figure 1 and Additional file 1: Table S1c). 64 of them presented large-scale alterations. This compilation shows the existence of highly recurrent chromosomal alterations, such as supernumerary chromosomes 8, 9, 12 and especially chromosome 14 (n = 25/64, for the latter) and partial or complete loss of chromosomes 1p, 13q, 16, 21 and particularly 22 (n = 34/64, for the latter). The compiled data also show the co-occurrence of chromosomal alterations, i.e. -1p/-22 (n = 5); +7/-16q (n = 5); +12/-22 (n = 6); −13q/-22 (n = 4); +14/-22 (n = 18). However, only the +14/-22 and the +7/-16q associations were non-random (p = 0,02 and p = 0,001, respectively, according to a two-tailed Fisher’s exact test). This suggests that the co-occurrence of +14/-22 and +7/-16q imbalances should confer a selective advantage, whose molecular basis remains to be elucidated.

Concerning the *FOXL2* locus, all tumors have kept the two alleles, although in two cases the DNA sequence displayed only the presence of the mutated version (data not shown). This can be due to either a second mutational hit or a gene conversion event that provides a selective advantage over heterozygous cells, as previously noted [14].

### Large-scale genomic alterations and their transcriptomic translation

**Figure 2:**
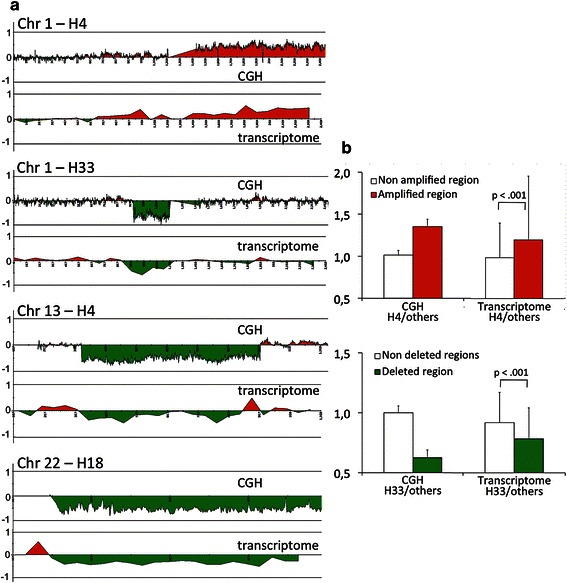
**Transcriptomic effects of large-scale genomic rearrangements in adult-type GCTs. a**. The CGH data (ratios tumor/reference) are displayed as log2 values averaged for sliding windows of 130 kb over the relevant chromosomes. For the transcriptomic data, we first computed the average expression levels for each gene (data from two transcriptomic hybridizations). Then we normalized gene expression as described in Methods. Normalized expression values were averaged over 30 windows (of the same size) per chromosome. Notice the close “correlation” between the chromosome copy-number and the expression levels of the genes involved in the imbalances. **b**. Comparison of the mean CGH values (ratios tumor/reference, in the natural scale) for the amplified Chr1q in H4 or the deleted segment of Chr1 in H33 with respect to the rest (non imbalanced) of the chromosome. For the transcriptome, the means of the normalized expression levels for genes located in altered segments (according to CGH) were significantly different from the means for genes located outside on the same chromosome (using both a *t*-test and a Mann–Whitney non-parametric test).

Next, we focused on the genes involved in the altered chromosomal segments and compiled their expression levels. Here, two transcriptomic hybridizations for the same tumor were combined and the average expression level for each gene was computed. Figure 2a shows that gene expression levels averaged over Mb-sized windows closely reflected the underlying chromosomal imbalances, as detected by CGH.

To further explore the influence of DNA copy number on gene expression, we compared the average expression of genes located in altered segments with that of genes located outside. For example, the copy number increased from 1.01 for the non-amplified segment of chromosome 1 in tumor H4 to 1.35 for the amplified region (Figure 2b). Consistently, the normalized gene expression averaged over the non-duplicated segment was 0.99 versus 1.21 for the duplicated region. A similar concordance was observed for other amplifications and deletions (Figure 2b and data not shown). Although there was a correlation between DNA amounts and mRNA levels, the degree of gene up- or down-regulation was always slightly lower. Although this effect might be due, in some cases, to contamination of tumor RNA with the transcriptome of neighboring normal cells, this explanation cannot apply to all samples. Thus, one is tempted to argue that some degree of expression compensation to chromosome dosage changes is taking place. Indeed, buffering of gene expression in response to genomic alterations have been reported in Drosophila harboring chromosomal imbalances [15-17], for human trisomy 21 [18] and for genes included in Copy-Number Variants (CNVs) [19].

### Identification of putative drivers: recurrently broken, amplified/duplicated or deleted genes

To further exploit our CGH and transcriptomic data, we focused on small-scale rearrangements that might help us pinpoint candidate genes whose duplication, deletion or breakage might be involved in tumorigenesis. First, we aimed at identifying amplified or deleted candidate drivers by combining GCH and transcriptomics. For this purpose, we generated a list of amplified or deleted CGH probes, whose log-ratio corresponded to at least 50% of the cells harboring a heterozygous duplication or deletion, in at least two tumors. Then, we computed the correlation coefficient (R) over all tumors between the CGH values and the mRNA expression values for the genes whose boundaries mapped at less than 25 kb from a copy-number-altered probe. This correlation filter was essential because a local genomic alteration does not necessarily imply a transcriptomic change. Thus, a meaningful driver, mapping to an amplified/deleted region, should display a reasonable correlation between copy number and mRNA expression. We set the threshold for statistical significance of Pearson’s correlation coefficient R to 0.63, which is the standard cut-off for ten samples. Genomic regions involved in large-scale imbalances such as trisomies or monosomies were analyzed separately by removing data from trisomic or monosomic tumors. For these regions, the threshold for R was adjusted to 0.67 or 0.71 in cases when 1 or 2 samples were removed. After excluding genes located within CNVs, we obtained a list of 48 candidates. After manual verification, we retained 13 amplified and 7 deleted genes fully located within the imbalances. Tumors harbored alterations ranging from 2 to 9/13 amplifications and from 1 to 7/7 deletions (Additional file 2: Table S2a).

A literature search shows the known or plausible implication in tumorigenesis for most of these 20 candidates (Table 1). *AKT1*, encoding a proto-oncogenic kinase, was the most frequently amplified gene (6/10 tumors). *AKT1* amplifications have been described in various types of cancer [20-22]. The second most frequently amplified gene (5/10 tumors) encodes the nuclear receptor NR1D1, a survival factor in a subset of breast cancers. Its driver effect might rely on its antiapoptotic activity [23] or on its known upregulation of genes involved in an abnormal aerobic glycolysis typical of cancer [24]. *MMAB*, found amplified in 4 out of 10 tumors, encodes the enzyme catalyzing the final step for conversion of vitamin B(12) into adenosylcobalamin. Interestingly, chemical derivatives of adenosylcobalamin are used to image breast, lung, colon, thyroid, and sarcomatous malignancies [25]. TSPAN32, amplified in 3/10 tumors, encodes a member of the tetraspanin superfamily, and is known to regulate cell proliferation. Along similar lines, another candidate encodes TENC1 (amplified in 2/10 tumors), known to stimulate PI3K/Akt signaling. Furthermore, *TENC1* knock-down decreases cell proliferation and its overexpression is associated with aggressive hepatocellular carcinoma [26,27]. This points to a deregulation of the PI3K/AKT pathway in GCTs, that would participate to tumorigenesis [28,29]. Another amplified candidate driver (3/10 tumors) encodes RANBP1, a cytoplasmic component of the nuclear pore complex. RANBP1 ensures cargo release from CRM1 upon export of specific mRNAs depending on the oncogenic factor eIF4E [30]. It is worth noting that these last two genes, along with 4 other amplified candidates, were found deleted in one tumor, H4, which was the only recurrence included in our samples.

**Table 1:** **Candidate driver genes identified as amplified or deleted in OGCTs, with correlated expression**

Chr	Gene	# of tumors with		Function & Implication in cancer if known	Ref
		Amp	Del		
14	***AKT1***	6	0	Known oncogenic kinase, core of one of the most frequently activated survival pathways in human cance.	[50]
17	***NR1D1***	5	0	Ligand-sensitive transcription factor, regulates the expression of core clock proteins; required for survival and proliferation of breast cancers	[24]
12	***MMAB***	4	0	catalyzes the final step for conversion of vitamin B(12) into adenosylcobalamin. Derivatives of the latter are used to image breast, lung, colon, thyroid, and sarcomatous malignancies.	[25]
11	***TSPAN32***	3	0	Membrane protein, regulates T cell proliferative responses. Tetraspanins are implicated in various steps of tumorigenesis.	[51]
11	***CTSW***	2	0	cysteine proteinase up-regulated in Large granular lymphocyte leukemia	[52]
12	***DGKA***	2	0	converts DAG into PA, a second messenger activating multiple signaling pathways implicated in tumorigenesis (i.e. mTOR signaling)	[53]
22	***RANBP1***	3	1*	Soluble component of the nuclear pore complex. Oncogenic overexpression of eIF4E induces overexpression of RANBP1	[30]
22	***TRMT2A***	3	1*	cell-cycle regulated protein, one of the 5 immunohistochemical markers in the Mammostrat test used to stratify breast cancers	[54]
12	***TENC1***	2	1*	Promotes PI3K/Akt signaling, KD = > decreased proliferation. High expression associated with aggressive hepatocellular carcinoma	[26]
16	***PIEZO1***	2	1*	transmembrane protein involved in mechanotransduction. Mediates integrin activation by recruiting R-Ras to the ER, modulating cell adhesion	[55]
12	***SPRYD3***	2	1*	SPRY domain containing 3. Not studied	na
22	***C22orf26***	3	2*	Not studied - now named *PRR34*, proline rich 34	na
22	***FAM19A5***	3	2	postulated to function as brain-specific chemokines or neurokines, acting as regulators of immune and nervous cells.	[56]
1	***NVL***	1*	3	AAA-ATPase, hTERT binding, essential for telomerase assembly. A nucleolar isoform is a component of pre-ribosomal particles	[36]
19	***C19orf18***	0	2	not studied. Identified as significantly binding to oligomeric β-amyloid	[57]
14	***FAM177A1***	0	2	Unknown function. Down-regulated by microRNA124 during neurogenesis. Identified as a target of the E3 ubiquitin-ligase FANCA.	[58]
12	***LIMA1***	0	2	Inhibits actin depolymerization and cross-links filaments in bundles. Putative suppressor of epithelial-mesenchymal transition and metastasis	[40]
17	***TADA2A***	0	2	transcriptional activator adaptor, in the PCAF and ATAC histone acetylase complexes, mediates DNA damage-induced apoptosis and G1/S arrest	[44]
5	***HSPA4***	0	3	Heat shock chaperone of the HSP110 family. Regulates cell proliferation and G1/S progression by releasing transcription factor ZONAB from tight junction sequestration	[59]
15	***RTF1***	0	3	part of the Paf1/RNA polymerase II complex, key regulator of transcription-related processes and cell-cycle progression	[34]

*These genes were found altered in the opposite way in the tumor H4, the only recurrent tumor in our cohort.

Among the recurrently deleted genes, *HSPA4*, deleted in 3 of the tumors, encodes a chaperone of the HSP110 family, predominantly expressed in the ovary [31]. Interestingly, *HSPA4* is known to regulate cell migration, both positively and negatively [32,33]. The second gene deleted in 3/10 tumors is *RTF1*, encodes a member of the Paf1 complex, which is a key regulator of RNA polymerase II transcriptional activity and of cell-cycle progression. RTF1 is critical for histone and chromatin modifications and telomeric silencing [34,35]. Another link with telomere maintenance is *NVL*, also found deleted in 3 tumors. *NVL* encodes an AAA-ATPase essential for hTERT binding and telomerase assembly [36]. In addition, a nucleolar isoform of NVL participates in ribosome biosynthesis [37]. *LIMA1* (a.k.a. *EPLIN*, Epithelial protein lost in neoplasm), deleted in 2/10 OGCTs, encodes a metastasis suppressor, frequently lost in cancer cells [38,39]. Consistently, it acts as a negative regulator of epithelial-mesenchymal transition and invasiveness [40] and its expression is inversely correlated with the aggressiveness of breast cancer [41]. Another interesting deleted gene, *TADA2A*, encodes an adaptor subunit of the PCAF and ATAC histone acetylase complexes. TADA2A-containing PCAF complex is essential for DNA-damage-induced acetylation of p53, necessary to promote cell cycle arrest and cell survival after DNA damage [42,43]. Moreover, TADA2A overexpression is pro-apoptotic in response to DNA damage [44]. Thus, its deletion in GCTs should provide resistance to apoptosis [45]. FOXO factors are known to be acetylated by PCAF upon stress to promote cycle arrest and DNA damage repair, or apoptosis. We have previously shown that FOXL2 is acetylated [46] and that it upregulates stress-response genes and induces cell-cycle slow-down [8]. A hyperlinked gene list with more complete information is provided in Additional file 2: Table S2a and S2b.

Next, we identified genes recurrently broken in the tumor samples. They were pinpointed by the existence, in at least 2 tumors, of one or several closely mapping breakpoints defined by amplifications/deletions upstream *and* downstream, within the relevant gene. A literature search for the 5 genes identified as broken showed that 4 of them are clearly involved in cancer (Table 2, more details are provided in Additional file 2: Table S2a and S2b). In particular, *NPAS3* and *RUNX1* are known tumor suppressors and *CELF4* is known to be frequently deleted in cancer. Broken genes might be fusion partners, as described for *RUNX1* in leukaemia [47], although we have no direct evidence for this.

**Table 2:** **Genes identified as broken in OGCTs**

Chr	Gene	Function & Implication in cancer if known	Ref
8	***C8orf34***	cAMP-dependent protein kinase regulator. Associated with irinotecan-related toxicities in patients with non-small-cell lung cancer.	[60]
18	***CELF4***	CELF/BRUNOL protein, alternative splicing factor. When lost, independent prognostic indicator in colorectal cancer.	[61]
14	***NPAS3***	Basic helix-loop-helix and PAS domain-containing transcription factor, tumor suppressor in astrocytomas	[62]
15	***SPG11***	Potential transmembrane protein phosphorylated upon DNA damage. Mutated in recessive hereditary spastic paraplegia.	[63]
21	***RUNX1***	CBF transcription factor subunit. Tumor suppressor, with oncogenic fusions in leukemias and mutations in breast cancers.	[64]

### The candidate drivers are expressionally clustered and share transcriptomic neighbors

**Figure 3:**
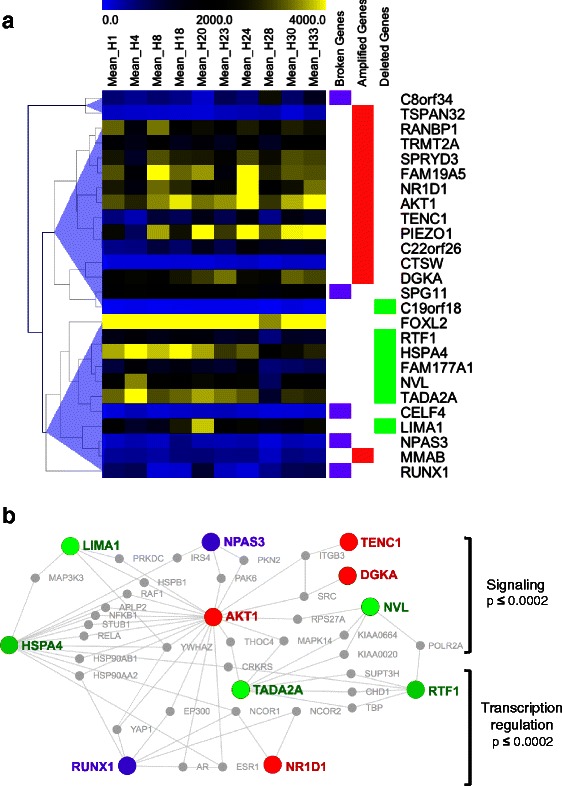
**Functional relationships between broken, amplified and deleted candidates drivers in adult-type GCTs. a.** Hierarchical clustering of the expression levels of the 5 broken genes (purple), 13 amplified (red), 7 deleted candidate drivers (green), and *FOXL2* in the 10 GCTs (see details in Methods). The clustering defines three groups of genes. The first group contains 5 deleted putative drivers together with the majority of broken genes, amplified *MMAB* and *FOXL2*. The second group includes almost all amplified genes, and one broken and 1 deleted genes. Amplified *TSPAN32* (anti-correlated to other amplified genes) defines a separate group along with the remaining broken gene. **b.** Physical interaction network involving the proteins encoded by broken, amplified or deleted candidate drivers and common binding partners. Known interactions were retrieved automatically by DAPPLE v2.0 (http://www.broadinstitute.org/mpg/dapple/dappleTMP.php, see [48]), using default parameters. The network was manually reorganized to highlight the expected hub position of *AKT1* and the partition of identified binding partners in signaling and transcription regulation. Gene set enrichment analysis was performed with Enrichr, for the 43 genes depicted in the network (the displayed p-values are Bonferroni-corrected).

To explore possible functional links among the candidate drivers, we performed a standard hierarchical clustering of the expression levels of the 20 amplified/deleted candidate drivers, the 5 broken genes and *FOXL2* (Figure 3A). This analysis defined three main groups: group 1 contained 6/7 deleted genes (i.e. *NVL*, *RTF1*, *TADA2A*, *HSPA4*, *FAM177A1, LIMA1*), 3/5 broken genes (which is coherent with a loss of function), 1 amplified gene (*MMAB*) and *FOXL2* itself; group 2 involved 11/13 amplified genes (including *AKT1*), 1 broken one and 1 deleted gene (*C19orf18*); and group 3 involved 1 broken gene (*C8orf34*) along with amplified *TSPAN32*. Functional links between these genes are supported by their interactions with common partners (Figure 3b), as detected by Dapple2 for 11/25 genes [48].

**Figure 4:**
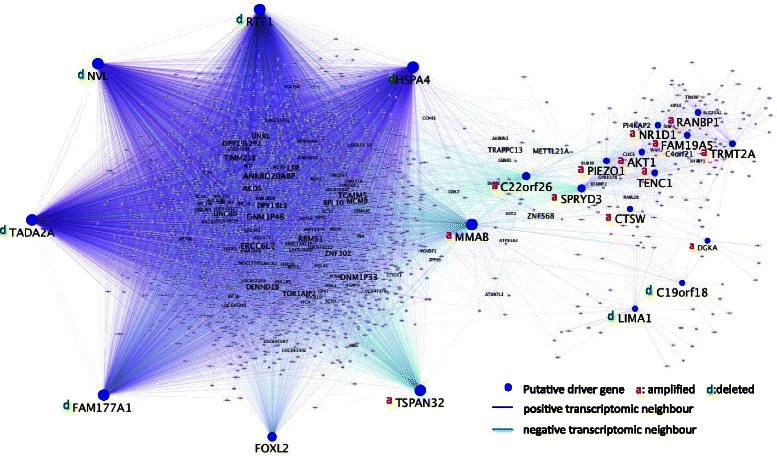
**Sharing of transcriptional neighbors among amplified/deleted genes.** The 20 putative drivers and *FOXL2* (blue nodes) are depicted within a network with strongly correlated transcriptional neighbors (R > =0,90), either positively (blue edges) or negatively (green edges). The diameter of the nodes reflects the number of neighbors. Amplified genes are labeled with a red a, and deleted ones with a green d. A high-resolution zoomable network is provided in Additional file 3: Figure S1. Notice that extensive sharing of transcriptomic neighbors parallels the same groups of candidate drivers than the expressional correlation in Figure 3a. Five of the deleted genes in the first group in Figure 3a share many positive and negative neighbors, and those neighbors are mainly negatively connected to the only amplified gene of this group, *MMAB*. The amplified genes of the second cluster (from Figure 2a) are grouped in a distinct sub-network with a connection to the dense sub-network of deleted candidate drivers restricted to *C22orf26* and *SPRYD3*. Amplified *TSPAN32* has a peculiar position, as it is connected only to neighbors of the first dense sub-network. Large grey nodes depict transcriptomic neighbors that are connected to a large portion of the candidate drivers (i.e. *POU3F1*, *MCM9*, *RPL10*, *POLR1D*, *PCNA*, *POLA1*, *PI4KAP2)*.

To further explore the implication of amplified/deleted candidate drivers in processes altered in cancer, we determined for each of them a list of positive and negative “transcriptomic neighbors” (i.e. genes whose expression levels displayed R > =0.63 or R = <−0.63 with the expression of the relevant driver over the tumor samples). Such transcriptomic (positive and negative) neighbors are more likely to be functionally linked to the drivers than random genes. A gene ontology analysis using EnrichR [10] showed that the negative transcriptomic neighbors of amplified drivers and the positive neighbors of deleted drivers were enriched in keywords involving cell cycle, suggesting a coherent effect of these two types of alterations. Other enriched keywords pointed to different types of cancer, DNA damage response, DNA repair and regulation of ubiquitinylation during mitosis (Additional file 2: Table S2c). Interestingly, all the candidate drivers shared a statistically significant proportion of transcriptomic neighbors. The network of Figure 4 (and Additional file 3: Figure S1) shows several interesting points: i) 5 of the deleted genes (*NVL, RTF1, TADA2A, HSPA4, FAM177A1*) included in group 1 share a highly significant number of transcriptomic neighbors and form a dense sub-network; ii) four amplified genes, *MMAB*, *SPRYD3*, *C22orf26* and *TSPAN32* are anti-correlated with a large number of positive neighbors of these 5 deleted putative drivers; iii) the amplified genes included in the expression group 2 also share transcriptomic neighbors and iv) *FOXL2* is heavily connected to the deleted putative drivers, which suggests an interplay between the C134W mutation and the genomic alterations (especially the deletions) that we have detected.

## Conclusions

In conclusion, our analysis identifies candidate co-driver genes, whose various alterations could contribute to GCT pathogenesis besides the FOXL2 somatic mutation. This is strengthened by their high degree of expressional interconnection, which suggests the existence of functional interactions among them, and by their known or suggested implication in cancer and related processes. However, we are aware that, given the small sample size for which CGH and transcriptomic data were available, this genomic exploration only provides leads for functional analyses to formally demonstrate the implication of the candidate drivers in GC tumorigenesis.

## Additional files

Additional file 1: Table S1. Tab a – Rainbow overview of genomic alterations in 10 adult ovarian GCTs. The CGH data is displayed as averaged values for sliding 130 kb windows, in log2 scale. In that scale, equivalent amount of genomic DNA for the tumor sample and the control DNA is displayed as values around the baseline at 0, segments below 0 indicate deletions, and segment above 0 indicate amplifications. Each chromosome is depicted with a different color. Tab b – Details of large-scale genomic alterations detected by CGH in the 10 GCTs. The clinical details were previously published in [49]. The recurrent alterations are indicated in bold. Tab c – Details of large-scale genomic alterations detected in ovarian GCTs by five different studies. The alterations are listed by chromosome. Clinical details are indicated when available. For each tumor, the alteration of the chromosome is written in black, other rearrangements present in the same tumors are indicated in grey and in parenthesis. Note that for acrocentric chromosomes, an alteration of the q arm is indicative of the amplification or loss of the whole chromosome (i.e. trisomy or monosomy). The last two columns highlight the associated recurrent alterations, and indicate whether these are statistically overrepresented (two-tailed p, Fisher’s exact test on a contingency table).
Additional file 2: Table S2. Tab a – List of broken, amplified and deleted candidate driver genes. This table is a more complete, detailed and hyperlinked version of Tables 1 and 2. Genes altered by breakpoint were identified by upstream and downstream rearranged CGH status in at least 2 tumors. The breakpoint must be within the gene in all the tumors, and not included in frequent CNVs detected in healthy control samples. Candidate driver genes were identified as presenting an expression significantly correlated (correlation coefficient >= 0.63) with the CGH status, in at least 2 tumors, without being included in CNVs detected in healthy control samples. CNVs were verified in DGV database, that contains the genomic alterations involving segments of DNA that are larger than >50bp identified in healthy control samples (DGV update: 31/05/2013). Tab b – Details of the genomic alterations leading to the identification of broken genes in ovarian GCTs. For each breakpoint are indicated: the tumors displaying an alteration, the genomic coordinates of the alterations, the name of the broken gene, bibliographic data centered on cancer-related processes, and the screenshot of the display in MeV software. Bright red and green regions are genomic segments respectively amplified or deleted in more than 50% of the cells in the tumor samples. Tab c – Keywords enrichment of transcriptomic neighbors lists for the 20 putative amplified/deleted drivers. For each candidate driver, the sets of transcriptomic neighbors with a statistically significant correlation of expression (R>= 0.63 or R<= −0.63) were tested using Enrichr. The keywords in KEGG pathways and in Gene Ontology Biological Process (GO-BP) are given for positive and negative neighbors, when they reached significant enrichment after correction of the p-value by the Bonferroni method. Cancer-related keywords are highlighted in red.
Additional file 3: Figure S1. High-resolution image of the network between the 20 candidate drivers and their highly-correlated transcriptomic neighbors. Blue nodes: 20 candidate drivers and *FOXL2*. Amplified genes are labeled with a red a, and deleted ones with a green d. Grey nodes: transcriptomic neighbors with expression correlated with a correlation coefficient >= 0.90 to at least one of the candidate drivers or *FOXL2*. The sizes of the node and of the node label are proportional to the number of edges. Some transcriptomic neighbors are connected to a large portion of the candidate drivers (large grey nodes). Purple edge: positive correlation between the candidate driver (or *FOXL2*) and its transcriptomic neighbor. Green edge: negative correlation between the candidate driver (or *FOXL2*) and its transcriptomic neighbor. The edges are rendered semi-transparent in order to keep the gene names visible. The picture is zoomable to see individual gene names. The Cytoscape session file is available upon request.
