# Supplementary figures and images for: Combined comparative genomic hybridization and transcriptomic analyses of ovarian granulosa cell tumors point to novel candidate driver genes
Source: BMC Cancer. 2015 Apr 10;15:251. doi: 10.1186/s12885-015-1283-0 (PMC4407711; doi:10.1186/s12885-015-1283-0)

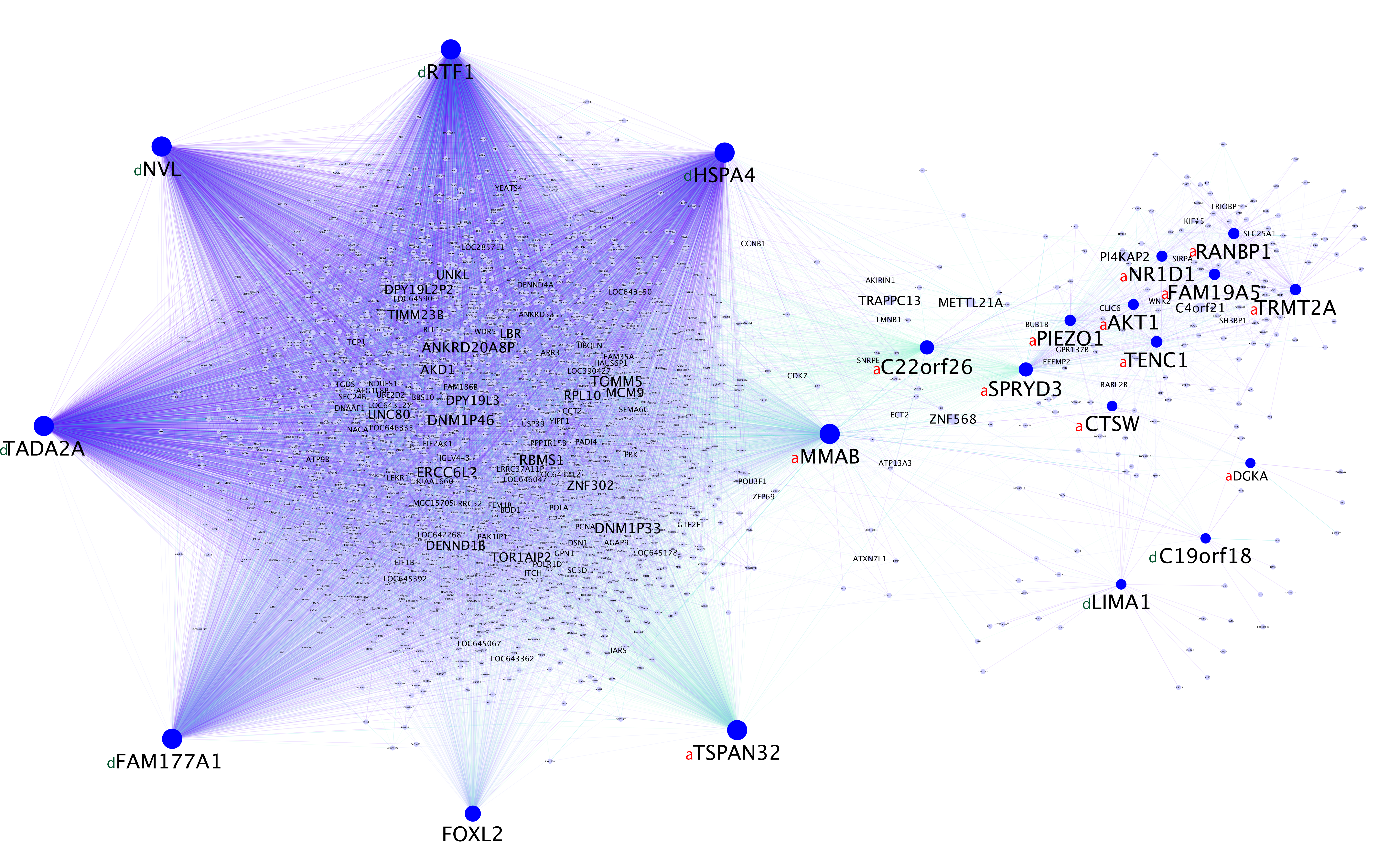

Supplement: Additional file 3: Figure S1. — High-resolution image of the network between the 20 candidate drivers and their highly-correlated transcriptomic neighbors. Blue nodes: 20 candidate drivers and FOXL2. Amplified genes are labeled with a red a, and deleted ones with a green d. Grey nodes: transcriptomic neighbors with expression correlated with a correlation coefficient >= 0.90 to at least one of the candidate drivers or FOXL2. The sizes of the node and of the node label are proportional to the number of edges. Some transcriptomic neighbors are connected to a large portion of the candidate drivers (large grey nodes). Purple edge: positive correlation between the candidate driver (or FOXL2) and its transcriptomic neighbor. Green edge: negative correlation between the candidate driver (or FOXL2) and its transcriptomic neighbor. The edges are rendered semi-transparent in order to keep the gene names visible. The picture is zoomable to see individual gene names. The Cytoscape session file is available upon request. [file 12885_2015_1283_MOESM3_ESM.png]
